# Insulin-like Growth Factor 1 Signaling Axis Meets p53 Genome Protection Pathways

**DOI:** 10.3389/fonc.2016.00159

**Published:** 2016-06-23

**Authors:** Haim Werner, Rive Sarfstein, Derek LeRoith, Ilan Bruchim

**Affiliations:** ^1^Department of Human Molecular Genetics and Biochemistry, Sackler School of Medicine, Tel Aviv University, Tel Aviv, Israel; ^2^Yoran Institute for Human Genome Research, Tel Aviv University, Tel Aviv, Israel; ^3^Diabetes and Metabolism Clinical Research Center, Rambam Health Care Center, Haifa, Israel; ^4^Department of Obstetrics and Gynecology, Hillel Yaffe Medical Center, Hadera, Israel

**Keywords:** insulin-like growth factor 1, IGF1 receptor, tumor suppressors, p53, BRCA1, transcription regulation, genome protection

## Abstract

Clinical, epidemiological, and experimental evidence indicate that the insulin-like growth factors (IGFs) are important mediators in the biochemical chain of events that lead from a phenotypically normal to a neoplastic cell. The IGF1 receptor (IGF1R), which mediates the biological actions of IGF1 and IGF2, exhibits potent pro-survival and antiapoptotic activities. The IGF1R is highly expressed in most types of cancer and is regarded as a promising therapeutic target in oncology. p53 is a transcription factor with tumor suppressor activity that is usually activated in response to DNA damage and other forms of cellular stress. On the basis of its protective activities, p53 is commonly regarded as the *guardian of the genome*. We provide evidence that the IGF signaling axis and p53 genome protection pathways are tightly interconnected. Wild-type, but not mutant, p53 suppresses *IGF1R* gene transcription, leading to abrogation of the IGF signaling network, with ensuing cell cycle arrest. *Gain-of-function*, or *loss-of-function*, mutations of p53 in tumor cells may disrupt its inhibitory activity, thus generating oncogenic molecules capable of *trans*activating the *IGF1R* gene. The interplay between the IGF1 and p53 pathways is also of major relevance in terms of metabolic regulation, including glucose transport and glycolysis. A better understanding of the complex physical and functional interactions between these important signaling pathways will have major basic and translational relevance.

## The Insulin-Like Growth Factor Network: Ligands, Receptors, and Binding Proteins

The processes of growth, development, and cell death are tightly regulated by multiple cellular and secreted factors that, in a highly orchestrated fashion, control the stage- and tissue-specific expression of a wide array of genes. Disruption of this finely tuned genetic program may lead to a pathological phenotype, including tumor formation. Modern biological research is aimed at dissecting physiological and pathological processes at defined levels of regulation and tackling biological questions in a comprehensive and integrated manner.

The insulin-like growth factors (IGF1 and IGF2) are a family of mitogenic peptides with important roles in diverse aspects of body function (1). The mature circulating IGF1 and IGF2 contain, respectively, 70 and 67 amino acids and display a marked similarity to proinsulin. Both peptides contain A and B domains, analogous to the A and B chains of insulin. However, unlike insulin, IGF1 and IGF2 retain the C-peptide, which is absent in the mature insulin molecule. For more than 50 years, the IGFs have attracted enormous scientific and clinical interest. This broad attention emanates from the appreciation that IGFs mediate fundamental biological processes at multiple ontogenetic stages (e.g., embryonic, infancy, adolescence, adulthood) and at virtually every level of organization (e.g., cells, tissues, organs, organisms) (2–5).

**Figure 1:**
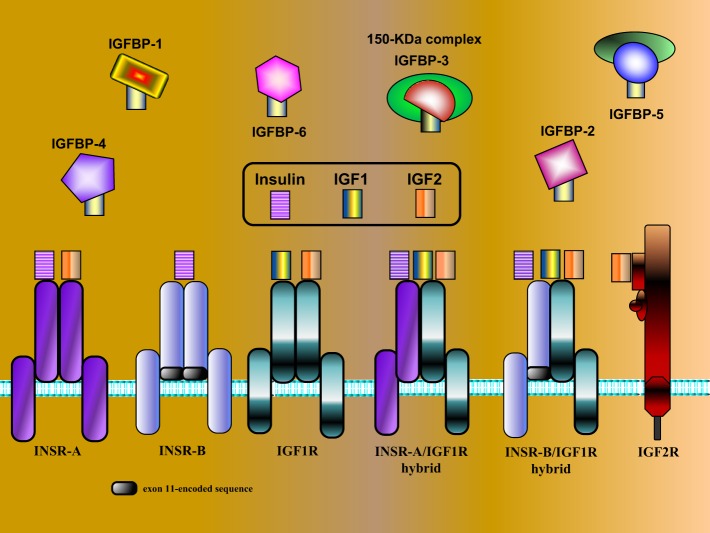
**Schematic representation of IGF network components**. The IGF system is comprised of three ligands (insulin, IGF1, and IGF2), three *typical* cell-surface receptors [insulin receptor (INSR), IGF1 receptor (IGF1R), and IGF2 receptor (IGF2R)], and at least six IGF-binding proteins (IGFBP1–6). The INSR has two isoforms, INSR-A and INSR-B, which differ in the absence or presence, respectively, of exon 11-encoded sequences. In addition to the typical receptors, naturally occurring hybrid receptors have been described in which an α/β INSR hemi-receptor is linked to an α/β IGF1R hemi-receptor. The IGF2R is a single-chain polypeptide composed of 15 repeat sequences and a short cytoplasmic domain. The IGF2R is homologous to the mannose 6-phosphate receptor and is involved in the recycling of lysosomal enzymes. IGFBP3 is the most abundant IGFBP in serum, and it is usually present as a ternary complex that includes the ligand and an acid-labile subunit (ALS). IGF bioavailability is also modulated by IGFBP proteases that cleave IGFBPs in a tissue-specific manner.

Insulin-like growth factors were originally identified as liver-secreted hormones, primarily involved in mediating the endocrine actions of growth hormone (GH, somatotropin) (6). Appropriately, the term *somatomedin* was initially adopted. Subsequently, the IGFs were conclusively demonstrated to be synthesized by most extrahepatic organs, sites in which they exhibit autocrine and paracrine modes of action. As explained in the next section, IGF1 is a progression factor that is needed to advance throughout the various phases of the cell cycle. The biological actions of IGF1 and IGF2 are mediated *via* activation of the IGF1 receptor (IGF1R), a transmembrane heterotetramer whose cytoplasmic tyrosine kinase domain is linked to the ras–raf–MAPK and PI3K–PKB/Akt signal transduction cascades (7–9). The IGF1R exhibits marked structural and functional homology to the insulin receptor (INSR), a finding that reflects a common evolutionary origin (10). The divergent biological activities of INSR and IGF1R are described in the next section. Unlike insulin that circulates in a free form in serum, IGF1 and IGF2 are bound in the circulation and extracellular spaces to a family of IGF-binding proteins (IGFBP1–6). IGFBPs control the bioavailability of both IGFs by modulating their release, transport, and degradation (11). The various components of the IGF network are illustrated in Figure 1.

## IGFs Play Key Roles in Homeostasis Regulation: Physiological and Pathological Aspects

The concept that INSR activation (mainly by insulin) leads primarily to metabolic activities while IGF1R activation (mainly by IGF1 or IGF2) leads to proliferative events was the prevalent dogma for more than 45 years (12, 13). These beliefs have been challenged in recent years due, in part, to the availability of transgenic and knockout animal models with organ-specific disruption or, alternatively, overexpression of IGF axis components. Whereas the general notion, in broad terms, is still regarded as correct, there is ample evidence in support of the view that INSR is also capable of mediating proliferative types of activities while, on the other hand, IGF1R is responsible for specific metabolic actions (14–16).

The structural and functional homology between INSR and IGF1R suggest that both molecules are derived from a common ancestral precursor that probably participated in food intake and regulation of cellular growth (17). A divergence of functions most likely occurred before the appearance of the first vertebrates (18). However, in view of their common evolutionary origins and semi-conserved architecture, there is a certain degree of cross talk between the insulin and IGF ligands and their receptors (19).

As mentioned above, IGF1 stimulates mitogenic responses and inhibits cell death in a wide variety of cell types (20). Quiescent cells in G_0_ can be induced to enter G_1_ by competence factors (e.g., PDGF, bFGF). Once the cell enters into G_1_, sub-physiological doses of IGF1 will allow the cell to evade arrest in G_1_ and to progress through the cell cycle (21), hence complying with the definition of progression factors (22). IGF1 exhibits a variety of cellular functions, including regulation of hormone synthesis and secretion, chemoattractant migration, immune cell recognition, and neuromodulation. Metabolic effects of IGF1 include, among others, elevation of glucose uptake and hypoglycemia, without lowering free fatty acid levels (1, 19). In addition, IGF1 improves renal function by increasing renal blood flow and glomerular filtration rate (23). The cardinal role of the IGF axis in growth and development was demonstrated by the severe growth deficits observed in mice in which components of the IGF system, each one individually and in combination, were disrupted by homologous recombination (24).

## The IGF1R: A Potent Cell Survival Mediator

Clinical and experimental studies conducted since the early 1980s have established that nearly all human tumors display augmented IGF1R concentrations (25, 26). Elevated receptor levels are correlated with enhanced IGF1 and IGF2 binding and amplified IGF1R activation (phosphorylation). However, the levels of expression of the *IGF1R* gene as a determinant of IGF action and, in particular, the pathological significance of IGF1R overexpression, are still open questions (27–32). The archetypal features of the IGF1R include the following.

[1] potent antiapoptotic and cell survival capacities;
[2] critical roles in invasion, metastasis, and angiogenesis;
[3] contribution to malignant transformation (4, 5).

Experimental corroboration in favor of a critical role for the IGF1R in oncogenesis was provided by studies showing that fibroblasts derived from IGF1R knockout mice, with a few exceptions, do not undergo transformation when exposed to cellular or viral oncogenes (33, 34). However, it is important to emphasize that IGF1R, *per se*, is not oncogenic. In other words, the ligand-activated receptor is unable to induce cellular transformation. Furthermore, high IGF1R levels do not necessarily reflect the existence of a malignant phenotype. Thus, reduced circulating IGF1 values, such as those associated with congenital IGF1 deficiencies, usually lead to IGF1R upregulation. However, there is no evidence that these elevated IGF1R concentrations are correlated with a malignant phenotype (28). As mentioned above, IGF1 functions as a progression factor capable of “pushing” cells, including *already transformed* cells, through the cell cycle. Clinical and experimental data are consistent with the view that IGF1R expression and activation are fundamental prerequisites for *acquisition* of a malignant phenotype (10, 35). The converse scenario (i.e., that enhanced *IGF1R* gene expression in cancer is a *consequence* of the malignant phenotype) is, similarly, a biologically plausible theory that merits consideration. The molecular mechanisms responsible for the regulation of *IGF1R* gene expression are described in the next section.

## Transcriptional and Epigenetic Regulation of the *IGF1R* Gene

Investigation of the *IGF1R* gene promoter is helping define the functional and physical foundations for transcriptional control of the gene. Comprehensive promoter analyses generated valuable information regarding *cis*-elements as well as *trans*-acting factors that are responsible for *IGF1R* gene expression under physiological and pathological conditions. Transcription rate of the *IGF1R* gene is primarily dependent on a number of stimulatory nuclear proteins, including zinc-finger protein Sp1 (36, 37), E2F1 (38), Krüppel-like factor-6 (KLF6) (39), high-mobility group AT-hook (HMGA1) (40), etc. Some of these transcription factors directly bind specific *cis*-elements, including arrays of GC boxes located in the proximal *IGF1R* promoter region (i.e., *protein*–*DNA* interactions), whereas other nuclear proteins interact with members of the basal transcription machinery (i.e., *protein*–*protein* interactions). Recent DNA affinity chromatography-based proteomic analyses using nuclear extracts of breast tumor cells along with biotin-labeled *IGF1R* promoter fragments, followed by mass spectroscopy, led to the identification of a large set of *IGF1R* promoter-binding transcription factors (41). These transcription factors fall into a number of functional categories:

[1] cytoskeleton-associated proteins;
[2] proteins involved in transcription and regulation of nucleic acid metabolism;
[3] proteins involved in nuclear stability, chromatin structure, cell cycle, and gene expression;
[4] proteins involved in DNA repair, breaking, replication, and cell death;
[5] proteins involved in RNA splicing and processing, and translation.

Hence, Bioinformatic analyses suggest that the *IGF1R* gene plays key roles in multiple, seemingly unrelated, cellular pathways.

The interplay between steroid hormones and the IGF1 axis is of major clinical relevance in specific types of cancer, in particular adult epithelial tumors with a strong endocrine background (42–44). In this context, nuclear receptors, such as the estrogen (ER) and androgen (AR) receptors, were shown to stimulate *IGF1R* gene expression *via* mechanisms that involve ER and AR binding to *IGF1R* promoter elements (45). On the other hand, mutant versions of AR were unable to enhance *IGF1R* promoter activity (46). Transcriptional regulation of the *IGF1R* gene by ER and AR is of major importance in the etiology of breast and prostate cancers, respectively (47, 48). In addition, the question whether DNA methylation is involved in epigenetic regulation of the *IGF1R* gene was recently investigated in a series of prostate cancer cell lines representing early or advanced (metastatic) stages of the disease. Results of methylation specific PCR, sodium bisulfite-direct DNA sequencing, and 5-Aza-2′-deoxycytidine experiments revealed that the *IGF1R* promoter is, most likely, not subject to DNA methylation at any stage of the disease (49).

Finally, a relevant question is whether genetic events might be linked to *IGF1R* overexpression in cancer. Most available clinical data support the notion that *IGF1R* gene mutations constitute a very rare event and no substantial evidence links *IGF1R* mutations and cancer. In fact, heterozygous *IGF1R* mutations were reported in cases of intrauterine and postnatal growth restriction, but not in association with cancer (28, 50). Similarly, *IGF1R* gene amplification has been reported only in a small number of breast cancer and melanoma cases, suggesting that this genetic event is not a common mechanism in malignancy.

## p53: A Key Player in Genome Integrity Protection

p53 is a transcription factor with tumor suppressor activity that typically accumulates in the cell in response to DNA damage (51). In its hyperphosphorylated state, p53 is capable of arresting cell cycle progression at the G_1_ phase. Mutation of the p53 tumor suppressor gene is the most common event in human cancer (52, 53). The p53 pathway is activated in response to a wide spectrum of cellular stress signals. These insults include DNA damage and telomere shortening, hypoxia, low nucleoside triphosphate pool sizes, spindle damage, heat and cold shock, inflammation and nitric oxide production, and, finally, activation of oncogenes by mutations (54, 55). These various strains bear the potential to decrease the fidelity of cell cycle progression and DNA replication, hence leading to increased mutation rates (56). Accumulation of mutations constitutes an early event in cellular transformation and may, eventually, lead to the establishment of a cancerous phenotype. p53-mediated cell cycle arrest enables damaged DNA to be repaired before the replicative phase of the cell cycle (53, 57). Alternatively, p53 can elicit an apoptotic program. Based on these important protective roles, p53 is commonly regarded as the “*guardian of the genome*.” As described below, there is solid experimental and clinical data that suggest that p53 interacts with the IGF1 pathway at a number of levels, including (1) transcriptional regulation of IGF axis components, including the *IGF1R* gene; and (2) convergence of cytoplasmic and nuclear IGF1 and p53 signaling pathways. Finally, evidence assembled in recent years indicate that, in addition to its well-documented capacity to govern cell cycle progression, p53 activation also has a major impact on metabolic processes, including glucose transport (58) and obesity (59). This topic is described below.

## Differential Regulation of *IGF1R* Gene Expression by Wild-Type and Mutant p53

**Figure 2:**
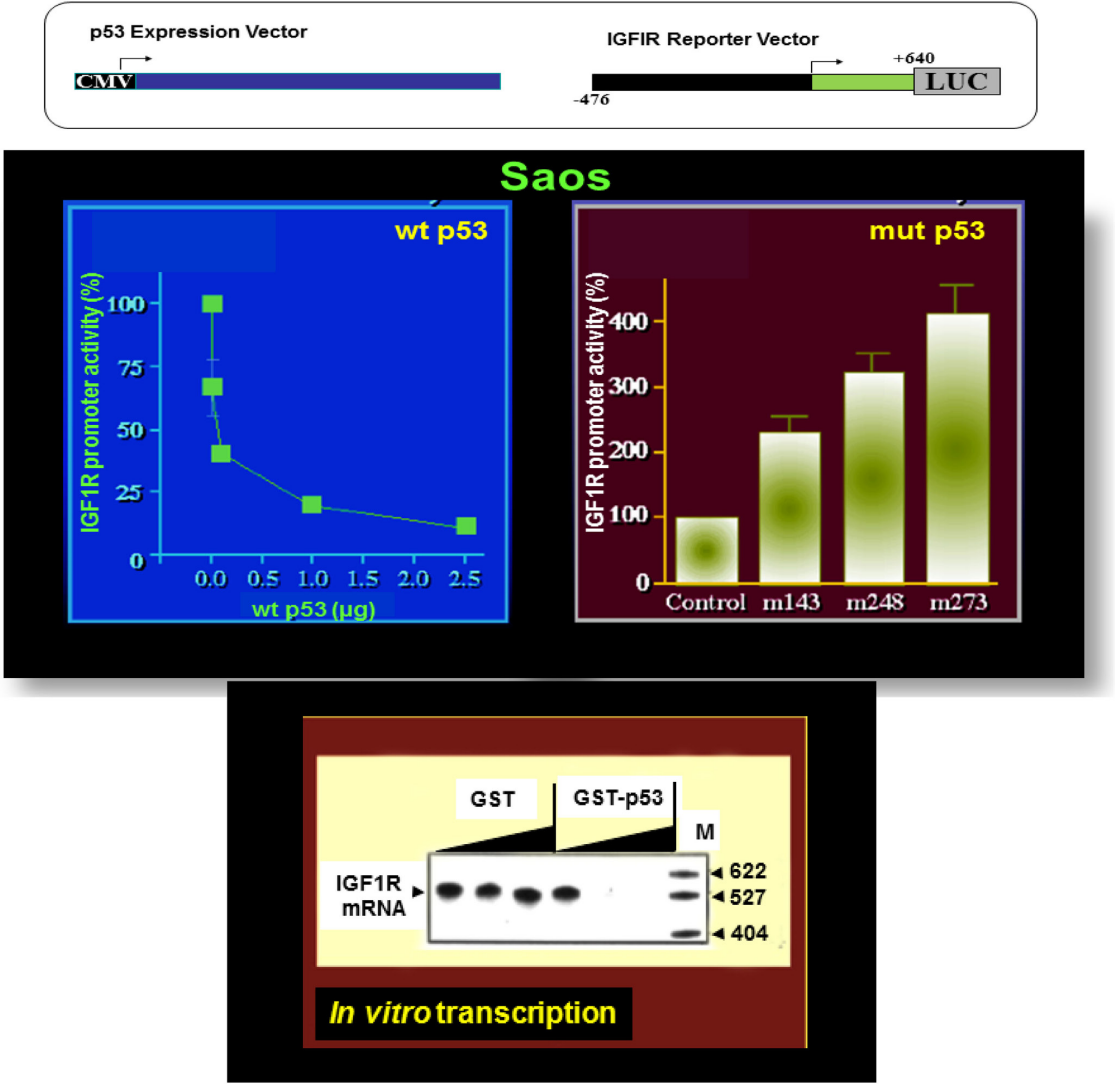
**Differential regulation of IGF1R gene expression by wild-type and mutant p53**. To evaluate the effect of p53 on *IGF1R* promoter activity, transient cotransfection experiments were performed in Saos-2 cells (an osteosarcoma cell line lacking endogenous p53) using a p53 expression vector along with an *IGF1R* promoter (nt −476 to +640) luciferase reporter construct (upper panel). Wild-type p53 suppressed IGF1R promoter activity in a dose-dependent manner, with maximal repression obtained with a dose of 2.5 μg DNA. Co-expression of tumor-derived mutant forms of p53 (bearing mutations at codons 143, 248, and 273) along with an IGF1R promoter led to transcriptional stimulation of the *IGF1R* gene. *In vitro* transcription assays using the purified wild-type p53 along with an *IGF1R* promoter template indicate that the effect of p53 was indeed mediated at the level of transcription.

The essential role of IGF1R in cell cycle progression and transformation led to the hypothesis that a potential mechanism by which the postmitotic, terminally differentiated cell kept out of the cell cycle may involve the constitutive inhibition of the *IGF1R* gene by wild-type forms of tumor suppressor genes (35). In accordance with this hypothesis, p53, the most frequently mutated tumor suppressor in human cancer, was identified as a *bona fide* negative regulator of the *IGF1R* gene. Cotransfection of a wild-type p53-encoding expression vector along with an *IGF1R* promoter luciferase reporter construct led to an ~90% suppression of *IGF1R* promoter activity (60, 61) (Figure 2). In contrast, tumor-derived mutant versions of p53-containing mutations at codons 143, 248, and 273 of the p53 molecule enhanced *IGF1R* promoter activity by 227, 319, and 406%, respectively.

**Figure 3:**
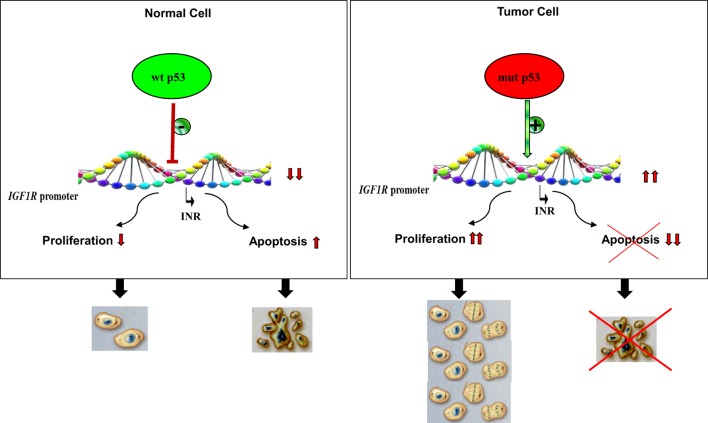
**Impact of p53 status on cell proliferation and apoptosis**. Under normal physiological conditions, activation of wild-type p53 following DNA damage, or other forms of cellular stress, may lead to stimulation or repression of various target genes. The *IGF1R* promoter has been identified as a *bona fide* target of p53. *IGF1R* transcription begins from a discrete promoter element termed *initiator* (INR). Transcriptional repression of *IGF1R* may lead to cell cycle arrest or, alternatively, increased apoptosis. p53 is the most frequently mutated gene in human cancer. Tumor cells containing mutant forms of p53 are typically expected to display large concentrations of IGF1R mRNA and cell-surface receptors. Increased IGF1 binding and IGF1R activation (phosphorylation) result in uncontrolled proliferation and/or abrogation of apoptosis, two critical traits of malignant cells.

Analyses aimed at defining the mechanistic basis of p53 regulation of *IGF1R* gene expression included, among others, transcription, mobility shift, and proteomic assays (41, 60, 61). Results of *in vitro* transcription assays using purified GST-p53 protein established that the tumor suppressor abolished transcription of an *IGF1R* gene template in a dose-dependent fashion (Figure 2). In addition, mobility shift assays indicate that p53 seems to exert its effects *via* protein–protein interactions with members of the basal transcription machinery, including the TATA-binding protein (TBP). The question whether p53 can directly bind *IGF1R* promoter DNA sequences is still under investigation. Proteomic analyses showed p53 binding to the *IGF1R* promoter, however, it is still unclear whether the tumor suppressor is part of a large multimeric protein complex or, alternatively, whether it binds *IGF1R* DNA in a sequence-specific manner (41). Combined data indicate that the mechanism of action of wild-type p53 involves transcriptional suppression of the *IGF1R* gene. *Gain-of-function*, or *loss-of-function*, mutations of p53 in tumor cells seem to disrupt its inhibitory activity, hence generating oncogenic molecules capable of transactivating the *IGF1R* gene. Because wild-type p53 is a potent inducer of apoptosis, we assume that the effect of p53 on apoptosis is mediated, at least in part, *via* suppression of the *IGF1R* promoter. Lack of *IGF1R* inhibition by mutant p53 molecules may help expand cancer cell populations that are otherwise destined to die (Figure 3).

**Figure 4:**
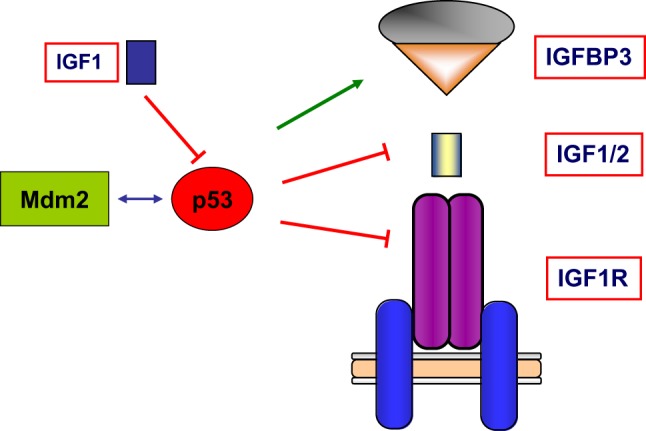
**Schematic representation of the interplay between the IGF1 signaling axis and p53 genome protection pathway**. p53 is activated in response to a broad spectrum of cellular insults and, in its hyperphosphorylated state, it can lead to cell cycle arrest and/or apoptosis. p53 was shown to inhibit transcription of the antiapoptotic genes *IGF2* and *IGF1R*. On the other hand, p53 enhanced expression of the proapoptotic gene *IGFBP3*. Thus, p53 is capable of regulating the activity of the entire IGF signaling axis by controlling expression and activity of ligands, receptors, and IGFBPs in a coordinated fashion. The abundance and activity of p53 itself was shown to be regulated by IGF1, which induced p53 degradation in an Mdm2-dependent fashion.

Finally, wild-type p53 was also shown to *inhibit* transcription of the antiapoptotic *IGF2* gene and to *enhance* transcription of the proapoptotic *IGFBP3* (62, 63). Hence, tumor suppressor p53 governs the activity of the entire IGF network by modulating in a coordinated fashion the expression of ligands, receptors, and binding proteins. A schematic representation of the interactions between the IGF axis and p53 is presented in Figure 4.

## Regulation of *IGF1R* Gene Expression by p53 Homologs

Studies have identified a family of proteins that are structurally and functionally related to p53. Members of this family, specifically p63 and p73, retain the basic features of the p53 protein, including an acidic, N-terminal transactivation domain, a central DNA-binding domain, and a C-terminal oligomerization domain (64–66). In addition, p63 and p73 exhibit some of the biological properties of p53, including the ability to recognize and bind p53 target sequences, transactivate p53-responsive genes, and induce apoptosis. However, unlike p53, the genetic structures of p63 and p73 are extremely complex, leading to the synthesis of several isoforms. To investigate whether novel members of the p53 family share the paradigm of p53 suppression of the *IGF1R* gene, the regulation of *IGF1R* by p63 and p73 was evaluated in colon cancer cells. Results of coexpression studies using the various p63 and p73 isoforms demonstrate that p63/p73 proteins suppressed *IGF1R* promoter activity in a dose-dependent manner, suggesting an anti-oncogenic role for p53 homologs (67). On the other hand, mutant proteins were impaired in their ability to inhibit the *IGF1R* gene.

Although the multiplicity of p63/p73 isoforms precludes any generalization regarding their roles in cancer biology, it is evident that this family of p53 homologs is involved in acquisition and maintenance of a malignant phenotype (64, 65). As depicted above for p53, negative regulation of *IGF1R* by p63/p73 leads to diminished IGF binding, a characteristic feature of terminally differentiated cells. On the other hand, disruption of p53/p63/p73 pathways in cancer cells may result in impaired suppression of *IGF1R* transcription, with enhanced binding and cell-surface receptor activation by endocrine or locally produced IGF1 and/or IGF2.

## Convergence of Tumor Suppressor p53 and IGF1 Signaling Pathways

Early studies suggested a potential convergence of the p53 and IGF1 signaling pathways (68). Binding of IGF1 to the IGF1R results in the recruitment and activation of PI3K to the plasma membrane receptor and activation of the Akt protein kinase. Akt has several antiapoptotic substrates, such as BAD and Mdm2 (69). Akt is translocated to the cell nucleus where it phosphorylates forkhead transcription factors, leading to antiapoptotic signaling and cell growth (70). PI3K activity is counteracted by PTEN, a lipid phosphatase. PTEN functions as a tumor suppressor and, similar to p53, is frequently mutated in breast cancer as well as other sporadic and familial malignancies (56). p53 regulates PTEN expression, while PTEN and inhibitors of Akt signaling upregulate p53 expression (71). As described below, the functional and physical connections between the IGF1 signaling pathways and tumor suppressor p53 take place at multiple levels of biological regulation (e.g., transcription, translation, stability, etc.) and occur in several cellular compartments (e.g., nucleus, cytoplasm).

The ubiquitin ligase Mdm2 is of primary importance in regulation of p53 activity (72), and IGF1 was shown to induce p53 degradation in an Mdm2-dependent manner (73). Girnita et al. have shown that Mdm2 physically associates with IGF1R and causes IGF1R ubiquitination and degradation (74). Mdm2 serves as a ligase in ubiquitination of the IGF1R and thereby causes its degradation by the proteasome system. Consequently, by sequestering Mdm2 in the cell nuclei, the level of p53 may indirectly influence the expression of *IGF1R*. This function of Mdm2 and p53 constitutes a potential mechanism for the regulation of IGF1R and cell growth. Various other mechanisms and signaling pathways have been suggested to participate in the convergent tasks of p53 and the IGF system and are thus regarded as potential therapeutic targets (75). This multifocal signal modulation therapy includes, in addition to the IGF1 axis and the Mdm2–p53 loop, additional targets, including the epidermal growth factor receptor, mammalian target of rapamycin, AR and ER, NFkB, etc.

An intact p53 signaling pathway is a critical prerequisite for many of the biological actions of IGF1. KLF6, a zinc-finger of the Krüppel-like family, was identified as a transcription factor involved in the regulation of genes associated with response to injury (76, 77). In addition, KLF6 plays a tumor suppressor role in colon and prostate cancer (78). IGF1 was shown to stimulate *KLF6* gene transcription in cells with normal, but not disrupted, p53 (79). These results identify the *KLF6* gene as a downstream target for IGF1 action and suggest that the mechanism of action of IGF1 requires an intact p53 pathway. An additional example of the critical need for p53 in IGF1 action is provided by folic acid, a member of the vitamin B family. Folic acid exhibits chemopreventive activity, and part of the antiproliferative action of this micronutrient can be attributed to its ability to inhibit *IGF1R* gene expression in a p53-dependent manner (80). The protective effect of folic acid was abrogated in cells lacking p53.

Of major interest, a recent study identified the *GH* gene as a direct transcriptional target of p53. Pituitary adenomas secreting GH are always benign and exhibit DNA damage and a senescent phenotype. The laboratory of Shlomo Melmed tested the effect of nutlin-induced p53-mediated senescence in pituitary cells (81). The authors showed that DNA damage induced by nutlin triggers the p53 senescent pathway, with subsequent induction of intracellular pituitary GH. In contrast, GH was not induced in p53-null cells. p53 was capable of binding specific *GH* promoter motifs and enhanced *GH* gene transcription and secretion in senescent pituitary adenoma cells. In summary, intracrine GH acts in pituitary cells as an apoptosis switch for p53-mediated senescence, likely protecting the pituitary adenoma from progression to malignancy. Given the key role of GH in regulation of IGF1 biosynthesis and secretion, the identification of the *GH* gene as a novel target for p53 action is consistent with the concept that the tumor suppressor constitutes an important systemic regulator of the *entire* GH–IGF1 endocrine axis.

## IGF1, IGF1R, and DNA Damage

Evidence accumulated in recent years revealed a strong link between the *IGF1R* gene and radiosensitivity, defined as cell killing after exposure to ionizing radiation. IGF1R overexpression in fibroblasts conferred radioresistance and, conversely, addition of antisense oligomers against IGF1R mRNA reversed the radioresistant phenotype (82). Immunohistochemical analysis of primary breast tumors indicated that high levels of IGF1R correlated with ipsilateral tumor recurrence following lumpectomy and radiation therapy. Of interest, the only growth factor receptor that provided protection from ultraviolet-induced apoptosis in keratinocytes was the IGF1R (83). It is conceivable that the activated IGF1R may, on one hand, function as a survival factor for irradiated cells and, on the other hand, induce a postmitotic state that prevents passage of damaged DNA to daughter cells.

The *ATM* (ataxia-telangiectasia mutated) gene encodes a 350-kDa protein whose mutation in ataxia-telangiectasia, a genetic neurological disorder, leads to progressive neuronal degeneration, premature aging, immunological abnormalities, and an increased risk of cancer (84). The central role of the ATM protein in signaling DNA damage is now well established. Ionizing, but not ultraviolet, radiation enhances ATM kinase activity and phosphorylates a series of target proteins, including p53 and BRCA1, which, as described here, are involved in cell cycle control and repair of DNA damage (85). The potential role of the *IGF1R* gene as a target in an ATM-dependent pathway involved in regulating the radiation response was inferred from studies demonstrating that IGF1R levels were reduced in cells carrying mutations in the *ATM* gene (86).

Complementation of mutant cells with the ATM cDNA resulted in increased *IGF1R* promoter activity and elevated IGF1R levels (87). Furthermore, forced expression of the IGF1R in ataxia-telangiectasia cells conferred increased radioresistance. Hence, data indicate that the *IGF1R* gene is a novel downstream target in an ATM-mediated DNA damage response pathway. The fact that p53 has been characterized as a target for ATM action suggests that the regulation of *IGF1R* by ATM is, most probably, a p53-dependent process. Deregulated expression of the *IGF1R* gene after ionizing radiation is linked to genomic instability and increased cancer rates.

Finally, the laboratory of Valentine Macaulay has shown that downregulation of IGF1R in melanoma cells was associated with enhanced radiosensitivity (88). The authors also showed that IGF1R depletion delays repair of radiation-induced DNA double-strand breaks (DSB) and demonstrated that IGF1R affects DSB repair by modulating both major DSB repair pathways, i.e., non-homologous end-joining and homologous recombination repair (20, 89, 90).

## Regulation of the IGF Axis by Genome Protection Genes: A Common Theme in Cell Biology

The identification of the *IGF1R* gene as a downstream target for members of the p53 family of genome protection genes led us to postulate the hypothesis that the expression and activity of the IGF1 axis, in general, and the *IGF1R* in particular, is governed by multiple families of negative regulators, i.e., tumor suppressor genes (35, 44). The rationale for this hypothesis lies in the fact that the *IGF1R* is usually overexpressed in tumors displaying *loss-of function* mutations of tumor suppressor genes (7–9, 26). In other words, our model proposes that the *IGF1R* gene constitutes a common downstream target for multiple tumor suppressors. While tumor suppressors might differ in their organ-specific expression, mechanisms of activation, type of tumors involved, and other parameters, they share the IGF1R pathway as a shared response path.

The breast and ovarian cancer susceptibility gene (BRCA1) is a transcription factor with well-defined roles in DNA damage repair, cell growth, and apoptosis (91, 92). Mutations in the *BRCA1* gene are detected in a large proportion of families with inherited breast and/or ovarian cancer (93, 94). BRCA1 mutation carriers have up to 87% estimated cumulative risk of developing breast cancer by age 70. Consistent with its tumor suppressor role, forced expression of BRCA1 in breast cancer cells led to a marked reduction in endogenous IGF1R levels and promoter activity (95–97). In contrast, a mutant *BRCA1* gene encoding a truncated version of the molecule (del185AG, a mutation with a high incidence among Ashkenazi Jews) had no effect on *IGF1R* expression. These results are consistent with the above postulated hypothesis, which proposes that the *IGF1R* gene is a downstream target for BRCA1 (and other tumor suppressors) action. Activation of BRCA1 in response to DNA damage, oxidative stress, or other cellular insults, may lead to a reduction in IGF1R levels and IGF action (98). Mobility shift assays performed with the full-length, *in vitro*-translated, BRCA1 failed to reveal binding of the protein to *IGF1R* promoter sequences. However, BRCA1 was shown to bind zinc-finger protein Sp1, a potent *IGF1R* gene transactivator, hence preventing it from binding to the *IGF1R* promoter.

A manifestation of the mechanistic interplay between BRCA1/BRCA2 and the IGF axis is seen in the clinics. Immunohistochemical analysis of IGF1R levels in breast tumor specimens derived from BRCA1/BRCA2 mutation carriers, compared to matched sporadic breast cancer patients, revealed higher IGF1R levels in tumors of BRCA mutation carriers (99). Furthermore, evidence in support of a complex interplay between the IGF1 axis and tumor suppressor BRCA1 was provided by studies showing that IGF1 increases *BRCA1* gene expression and enhances *BRCA1* promoter activity (100). Inhibitory control of *IGF1R* gene expression by BRCA1 may constitute a protection mechanism that prevents from normal breast cells from engaging in mitogenic activity. Lack of *IGF1R* inhibition by mutant BRCA1 may lead to enhanced *IGF1R* levels, an important prerequisite for malignant transformation (101).

Additional examples of tumor suppressor genes whose mechanisms of action involve transcriptional suppression of the *IGF1R* gene are the von-Hippel–Lindau (VHL) protein, a gene with important roles in the etiology of renal cancer (102), and WT1, a zinc-finger tumor suppressor with key roles in Wilm’s tumor, or nephroblastoma (103, 104). Of cardinal importance, the ability of WT1 to suppress *IGF1R* transcription is strictly dependent on the cellular status of p53. Thus, WT1 exerts its inhibitory role *only* in cells expressing a wild-type p53 gene, whereas it is unable to repress the *IGF1R* gene in cells with a mutant p53.

In summary, the *IGF1R* gene constitutes a common target for multiple oncogenes and antioncogenes. Inhibition of IGF1R expression and activation by negative growth regulators is expected to keep IGF1R levels below a certain threshold. We assume that low IGF1R levels are, for the most part, incompatible with the execution of mitogenic activities (Figure 3). p53 displays both direct and indirect roles in regulation of the *IGF1R* gene by virtue of its capacity to modulate the transcriptional activities of multiple transcription factors.

## Can p53 Status Predict Responsiveness to IGF1R-Directed Targeted Therapies?

Given its strong pro-survival activity along with its universal expression in cancer cells, the IGF1R emerged in recent years as a promising therapeutic target in oncology (105–107). Unfortunately, results of phase I/II clinical trials have shown variable responses to IGF1R-directed therapies. The reasons for the failure to translate solid experimental and preclinical data into the clinics are complex and not fully explored. However, it has been suggested that most clinical trials using IGF1R inhibitors were conducted on unselected patients, and this fact had a negative impact on trials outcome. Therefore, identification of biomarkers that can predict response to targeted therapy is a major goal in current cancer treatment (108, 109).

While in certain cancers, including Ewing’s and rhabdomyosarcoma, tumor IGF1R levels were correlated with responsiveness to IGF1R-directed therapies, most clinical data seem to indicate that IGF1R expression levels, *per se*, do not predict sensitivity to IGF1R inhibition (110). Among a number of potential scenarios, it has been suggested that the specific molecular context and the complexity of the IGF1R–INSR hybrid receptors formation may explain part of the contradictory results. Likewise, circulating ligand and/or IGFBP levels are not always associated with the degree of anti-IGF1R effectiveness. Regarding the impact of IGF1R subcellular distribution on tumor phenotype and responsiveness to IGF1R-directed therapies, Aleksic et al. (111) reported the presence of nuclear IGF1R in primary renal cancer cells, formalin-fixed tumors, preinvasive lesions of the breast, and rapidly proliferating non-malignant tissues, and they also demonstrated that nuclear IGF1R was associated with poor prognosis in renal cancer. Asmane et al. (112) performed an immunohistochemical analysis of nuclear IGF1R in patients with unresectable or metastatic soft tissue sarcomas, Ewing’s sarcoma, and osteosarcoma treated with anti-IGF1R. In contrast to the previous study, exclusive intranuclear IGF1R presence was correlated with a better progression-free survival. Further studies are expected to shed light on this emerging aspect of IGF1R biology.

The impact of *p53* mutational status on selective IGF1R-targeted therapies is of major translational relevance. A recent study has shown that picropodophyllin, an IGF1R inhibitor of the cyclolignan family, prevented the growth of wild-type, but not mutant, p53-expressing colorectal carcinoma cell lines (113). Likewise, cixutumumab, an IGF1R monoclonal antibody, inhibited proliferation of a uterine papillary serous carcinoma cell line expressing a wild-type p53 gene but had no effect on uterine cells containing a mutant p53 (114). In conclusion, the potential role of p53 as a biomarker for IGF1R-directed therapies in human cancers must be confirmed by large cell-based and patients’ analyses.

Finally, a number of obstacles must be resolved in order to translate the success of preclinical studies into the clinical setting. These difficulties are primarily due to the considerable similarity between the mature forms of IGF1R and INSR. The homology between the tyrosine kinase domains of both receptors reaches 84%, and the downstream pathways elicited by IGF1R and INSR are almost identical (14). The possible effect of IGF1R-targeted therapy on INSR action is of major concern. Impaired insulin signaling in classical insulin target organs (e.g., muscle, adipose tissue, etc.) may lead to metabolic complications, including development of insulin resistance. On the other hand and given the well-documented involvement of INSR in various types of cancer, in particular breast tumors, experts in the field advise the combined targeting of both IGF1R and INSR.

## IGF1 and p53 Collaborate in Regulation of Metabolism

In addition to the well-established interplay between the IGF1 and p53 signaling pathways in the context of cell survival, proliferation, and cancer, as described above, evidence is mounting in support of novel homeostatic and metabolic activities of p53. Since its discovery in the mid-1950s, the insulin-like activities of IGF1 have been particularly well described. IGF1 has direct effects on fuel metabolism and, when given acutely, it enhances glucose uptake by muscle and abrogates hepatic glucose production (1). p53 has an important role in promoting oxidative phosphorylation (115). In addition, p53 has been shown to contribute to mitochondrial mass, a parameter of energetic expenditure (116), and cells expressing the tumor suppressor were shown to derive a larger portion of their ATP from oxidative phosphorylation, in comparison to cells devoid of p53 (117). Further evidence of p53 involvement in energy balance arises from the observation that, in addition to its nuclear localization, p53 is also present in mitochondria (118). It has been suggested that the mitochondrial presence of p53 in resting cells attests to the involvement of the tumor suppressor in normal mitochondrial activity.

p53 has an important role in the regulation of glycolysis. Most experimental evidence seems to indicate that, in agreement with its tumor suppressor role, p53 is capable of lowering glycolysis (119, 120). This activity can be regarded as an attempt by p53 to counter the acquisition of aerobic glycolysis usually associated with cancer cells (115). However, this intuitive rationalization is obscured by the finding that enhancement of glycolysis has also been associated, under certain circumstances, with p53 action. Of major interest, p53 has been identified as an important regulator of glucose transport, and wild-type p53 was shown to repress transcription of both *GLUT1* and *GLUT4* promoters in transfection assays (32). The inhibitory effect of wild-type p53 was abolished when cells were transfected with tumor-derived mutant versions of p53, harboring mutations in codons 143, 248, or 273. Evidence in support of a direct effect of p53 was provided by results of mobility shift assays showing binding of p53 to *GLUT4* promoter sequences. These results suggest that the ability of p53 to prevent tumor initiation is, in part, mediated by its ability to inhibit glucose uptake and cell energy supply. In contrast, mutant p53s have an impaired capacity to repress *GLUT1* and *GLUT4* promoters. Of interest, p53 was also shown to promote the expression of gluconeogenesis-related genes (e.g., G6PC, PCK2, etc.) and to enhance hepatic glucose production (121). By facilitating glucose export, p53 may prevent it from being shunted to proliferative pathways, such as glycolysis.

In the context of lipid metabolism, both IGF1 and p53 have been linked to sterol regulatory element binding protein (SREBP) activity. SREBPs are transcription factors that bind to sterol regulatory element DNA sequences, leading to enhanced synthesis of enzymes involved in sterol biosynthesis. SREBPs are required for cholesterol and fatty acid biosynthesis. p53 was shown to be induced in adipocytes of *ob/ob* mice, leading to inhibition of SREBP-1 and lipogenic genes (122). Luciferase assays revealed that p53 expression suppressed the promoter activity of the *SREBP-1c* gene. Hence, p53 activation may constitute a negative feedback loop with important roles in prevention of fat accumulation in adipocytes. On the other hand, IGF1 was shown to stimulate SREBP expression and lipogenesis *via* activation of the PI3K pathway (123). Taken together, the interplay between the IGF1 and p53 pathways may have major implications in lipid metabolism and, probably, obesity.

## Concluding Remarks

Overexpression of the *IGF1R* gene constitutes a common trait of many, if not most, tumors. The expression of the *IGF1R* gene is determined, to a large extent, at the transcriptional level, and the *IGF1R* promoter has been identified as a molecular target to a family of stimulatory transcription factors as well as nuclear proteins with tumor suppressor activity. Tumor suppressor p53 is the most frequently mutated molecule in human cancer. p53 is capable of arresting cell cycle progression at the G_1_ phase, thus enabling damaged DNA to be repaired before the replicative phase of the cell cycle. Alternatively, p53 can elicit an apoptotic program. In the present review article, we provided evidence that the mechanisms of action of wild-type p53 and p53 homologs (e.g., p63, p73) involve transcriptional suppression of the *IGF1R* gene. *Gain-of-function* or *loss-of-function* mutations of p53 disrupt its inhibitory activity and generate potentially oncogenic molecules capable of *trans*activating the *IGF1R* gene. While the interplay between the IGF1 signaling axis and the p53 genome protection pathways has been primarily investigated in the context of cancer cells, new evidence links these converging networks to the regulation of metabolism, including glucose transport, glycolysis, mitochondrial biology, and energy generation. Dissection of these complex regulatory loops will have a major impact on our comprehension of basic physiopathological processes as well as on our ability to personalize cancer therapies.

## Author Contributions

IB and HW: substantial contributions to the conception and design of the work and interpretation of data for the work; drafting the work and revising it critically for important intellectual content; final approval of the version to be published; and agreement to be accountable for all aspects of the work in ensuring that questions related to the accuracy or integrity of any part of the work are appropriately investigated and resolved. RS and DL: substantial contributions to the design of the work and interpretation of data; revising it critically for important intellectual content; final approval of the version to be published; and agreement to be accountable for all aspects of the work.

## Conflict of Interest Statement

The authors declare that the research was conducted in the absence of any commercial or financial relationships that could be construed as a potential conflict of interest.
